# Surgical and Systemic Treatment of Hereditary Breast Cancer: A Mini-Review With a Focus on BRCA1 and BRCA2 Mutations

**DOI:** 10.3389/fonc.2020.553080

**Published:** 2020-10-20

**Authors:** Athanasios Pouptsis, Leyla Swafe, Maneesha Patwardhan, Chara Stavraka

**Affiliations:** ^1^Department of Medical Oncology, Euromedica General Clinic of Thessaloniki, Thessaloniki, Greece; ^2^Department of Surgery, Queen Elizabeth Hospital, Lewisham and Greenwich NHS Trust, London, United Kingdom; ^3^Department of Surgery, King’s College Hospital NHS Foundation Trust, London, United Kingdom; ^4^Department of Medical Oncology, Guy’s and St Thomas’ NHS Foundation Trust, London, United Kingdom

**Keywords:** genes, hereditary, breast cancer, BRCA1, BRCA2, surgical management, systemic treatment

## Abstract

Hereditary breast cancer accounts for 5%–10% of breast cancer cases. The majority of familial cases have been linked to germline mutations in BRCA1 and BRCA2 genes, though other high penetrance susceptibility genes have also been identified through genomic testing advances. Optimal surgical treatment for these patients, who are of a younger age, has several challenges as it usually involves aggressive therapeutic and risk reducing interventions. At the same time, the therapeutic armamentarium for BRCA1/2 mutation carriers apart from platinum salts, has been enriched with the addition of poly-ADP ribose polymerase (PARP) inhibitors with promising outcomes. In this review we provide a succinct and comprehensive overview of the surgical and systemic treatment options for patients with BRCA1/2 mutation related breast cancer and an update on the most recent systemic treatment advances.

## Introduction

Breast cancer (BC) is the most common female malignancy, with more than 2 million cases being diagnosed world-wide annually (1). Hereditary syndromes account for approximately 5-10% of the cases and are associated with the presence of germ-line mutations. The majority of hereditary breast cancer cases result from mutations in BRCA1 and BRCA2 genes, whereas the rest have been linked to less frequent germline mutations in other high penetrance genes such as TP53, STK11, PTEN, CDH1, and PALB2, as well as moderate penetrance genes like ATM and CHEK2 (2).

Both BRCA1 and BRCA2 are tumour suppressor genes encoding proteins involved in homologous recombination repair (3). Pathogenic variants in both genes affect 1 in 400 persons in the general population and 1 in 40 in the Ashkenazi Jewish population. They get inherited by an autosomal dominant pattern and carry a lifetime cumulative breast cancer risk of 72% for BRCA1 and 69% for BRCA2 (4).

This review will focus on the surgical and systemic treatment of hereditary breast cancer with a particular focus on BRCA1 and BRCA2 mutations.

## Surgical Treatment

### Surgery on Locoregional Disease

The optimal surgical treatment for operable BC in BRCA1/2 mutation carriers depends on several factors and remains a topic of debate. Although breast conserving surgery (BCS) is the preferred surgical treatment for early stage disease in sporadic breast cancer, its oncological safety in BRCA1/2 mutation carriers has not been extensively studied. A meta-analysis of 10 studies, demonstrated a significantly higher risk for ipsilateral breast recurrence (IBR) in BCRA1/2 mutation carriers compared to non-carriers following BCS at a median follow up greater than 7 years, but no difference for shorter follow up periods (5). The risk for contralateral breast cancer was also found to be increased in BRCA1/2 mutation carriers (5). Although BCS is associated with higher IBR risk compared to mastectomy in BRCA1/2 mutation carriers, no difference was found between the two treatment options for overall survival, breast cancer death, or distant recurrence **(****Table 1****)** (5–8). Data from a meta-analysis indicate that the risk of IBR in BRCA1/2 mutation carriers who have undergone BCS was found to be reduced with adjuvant chemotherapy (RR 0.51, 95%CI 0.31–0.84) and oophorectomy (RR 0.42, 95%CI 0.22–0.81) (5).

**Table 1:** Summary of main studies investigating the role of breast conserving surgery and risk reducing mastectomy in breast cancer patients with BRCA1/2 mutations.

Author, year	Study design	Endpoints	Outcome
Nilsson et al. (6)	Retrospective cohort study Women with stage I-III BC N=162, BCT=45/M=117 Comparison between BCT vs mastectomy in BRCA1/2 carriers	LR as first recurrence Death resulting from BC Overall survival Distant recurrence	Increased risk for LR in BCT (new primary in most cases) No difference in BC related death, overall survival or distant recurrence
Pierce et al. (7)	Retrospective cohort study N= 655, BCT=302/M=353 Comparison between BCT vs mastectomy in BRCA1/2 carriers	LR as first recurrence Development of CBC BC specific survival	LR more likely in BCT but in 70% of cases new primary No difference in CBC or BC specific survival
van den Broek et al. (8)	Retrospective cohort study *BRCA1* (N = 191) and *BRCA2* (N = 70) Non carriers= 5820 Comparison between BCT vs mastectomy in BRCA1/2 carriers and non carriers	Ipsilateral recurrence Overall survival BC specific survival	No difference between BRCA1 carriers (10-year risk = 7.3%) and noncarriers (10-year risk = 7.9%) No difference in OS or BC specific survival Data for BRCA2 carriers insufficient for conclusions
Evans et al. (9)	Observational study N=718 patients with UBC BRCA1 (N=357)/BRCA2 (N=361) Comparison between N= 105 who underwent CRRM to controls with no CRRM: 593 carriers and 105 specifically matched	Overall survival	CRRM group 10-year survival: CRRM only: 83% CRRM + RRBSO: 92% Non CRRM group 10-year survival: No RRBSO:65% + RRBSO: 81% CRRM appears to confer a survival advantage. But warrants prospective validation in larger cohort.
Heemskerk- Gerritsen et al. (10)	Prospective cohort study N=583 patients with BRCA-associated BC. CRRM: N=242 patients (42%) underwent Surveillance: N 341 patients (58%) Examined efficacy of CRRM on OS	Overall survival (measured in PYO) CBC Incidence	CRRM: 4 patients developed CBC (2%). Surveillance: 64 patients developed CBC (19%) OS: 8% of patients died in CRRM group; 16% in the surveillance group Mortality was lower in CRRM group, 21.6 vs. 9.6 per 1000 PYO (Adjusted HR=0.49, 95% CI 0.29-0.82)
Metcalfe et al. (11)	Retrospective cohort study N= 390 BRCA1/2 carriers with EBC Unilateral M: N= 309 Bilareral M: N= 181	Breast cancer related death	Survival rate at 20 years: Contralateral M: 88% (95% CI, 83% to 93%) Unilateral M: 66% (95% CI, 59% to 73%)
van Sprundel et al. (12)	Retrospective cohort study N= 148 women with a BRCA1 or BRCA2 previously treated for invasive stage I-III BC N=79 opted for CRRM N=69 women remained under close surveillance. Mean follow-up was 5 years post diagnosis	Risk of CBC CBC specific and overall survival	CRRM: One case (1.3%) of invasive CBC Surveillance: 6 cases (14%) (P < 0.001) Risk of CBC in CRRM group vs. risk of CBC in surveillance group HR=0.09 (95% CI 0.01-0.78) P=0.03 Breast cancer-specific survival not significantly better in CRRM group (P=0.11) At 5 years follow-up, OS was 94% in CRRM group vs 77% in the surveillance group (P=0.027). With adjustment for BPO, CRRM group did not have significantly better survival vs. surveillance group HR 0.35, P=0.14

BCS could be considered a safe and reasonable option for BRCA1/2 mutation carriers but this should be discussed on an individual basis and further factors need to be taken into account. These include patient’s understanding of the increased risk for an ipsilateral new primary breast cancer with all potential emotional implications, as well as their ability to undergo appropriate breast surveillance.

International guidelines recommend that early breast cancer patients carrying mutations in moderate penetrance breast cancer susceptibility genes, should be offered BCS if appropriate. However, patients carrying TP53 germline mutations should avoid BCS followed by radiation as they are at high risk of developing radiation induced malignancies such us angiosarcoma (13).

### Risk Reducing Mastectomy

The term “risk reducing” has been deemed more appropriate than “prophylactic” in recent times as no mastectomy can remove all breast tissue. Several studies demonstrated a reduction in the risk of breast cancer by ~95% in BRCA1/2 mutation carriers who underwent bilateral risk reducing mastectomy (BRRM) in combination with oophorectomy and by ~90% in those with intact ovaries (14–17). A recent systematic review confirms the benefit of BRRM in reducing both incidence and mortality from breast cancer in high risk patients, such as BRCA1/2 carriers, but calls for rigorous prospective studies due to methodological flaws of the existing literature (18). Data for contralateral risk reducing mastectomy (CRRM) for patients who have had breast cancer in one breast are less conclusive as existing studies show a reduction in the incidence of contralateral breast cancer but no definitive survival benefit **(****Table 1****)** (9–12, 18).

For high risk patients such as BRCA1/2 mutation carriers, international guidelines recommend RRM with appropriate counselling on risks and benefits. When assessing the risk for developing contralateral breast cancer (CBC) the following factors need to be taken into account: age at diagnosis of primary breast cancer, family history, ability to undergo indicated surveillance imaging, prognosis from this or other malignancies, comorbidities and life expectancy (13, 19). RRM cannot completely eliminate the risk of breast cancer and can have a negative impact on body image and quality of life due to potential complications such as multiple surgeries, chronic pain, sexual dysfunction and poor cosmetic outcomes (20). Women considering this procedure should be well informed and weigh the risks and benefits compared to other alternatives such as risk reducing bilateral salpingo-oophorectomy, chemoprevention and intensive screening. For women who wish to avoid or delay RRM, MRI-based breast screening is a reasonable option (19, 21). For patients who undergo RRM, skin sparing mastectomy with or without preservation of the nipple-areolar complex has been found to be a safe option for BRCA carriers while achieving better cosmesis (22, 23).

There is a lack of data in the existing literature on the risk for CBC in breast cancer patients carrying mutations in cancer susceptibility genes other than BRCA1/2. Limited data exist for the CHEK2 1100elC frameshift mutation which is associated with a 3-fold increase in the risk of CBC (24). Decisions on CRRM for patients with moderate risk mutations should not be extrapolated from existing data on BRCA1/2, but should be balanced on several factors (age at diagnosis of primary breast cancer, family history, ability to undergo surveillance imaging) and involve appropriate patient counselling (13).

### Risk Reducing Bilateral Salpingo-Oophorectomy

Risk reducing bilateral salpingo-oophorectomy (rrSBO) is recommended for female BRCA1/2 carriers who have completed childbearing and should be completed by age 35 to 40 for BRCA1, 40 to 45 for BRCA2 carriers or earlier as per patient’s relevant family history (25). It has been demonstrated that rrBSO reduces the risk for ovarian cancer by 80% and all-cause mortality by 68% in female BRCA1/2 carriers (26, 27). The beneficial effect of rrBSO on breast cancer risk reduction has also been assessed but current data are less conclusive. Some prospective studies confirmed that rrBSO reduces BC risk for both BRCA1/2 carriers (25, 28). However, a large case-control study showed a benefit for rrBSO only for BRCA1 carriers when performed before the age of 40, while a more recent study identified a benefit only for BRCA2 carriers when performed prior to 50 years old (29). Oophorectomy has been associated with a significant decrease in the risk of IBR and CBC (5).

## Systemic Treatment

Germline mutations of BRCA1 and BRCA2 genes lead to the decreased capacity of the cell to repair double strand breaks (DSBs), as they are key elements of the homologous recombination (HR), one of the two main mechanisms of DSB repair (30, 31). This formed the basis for the development of new therapeutic strategies and the development of novel treatments for this specific breast cancer patient subgroup (**Table 2**).

**Table 2:** BRCA1/2 associated breast cancer and systemic treatment.

Authors, year	Phase	Treatment	Setting	Endpoint	Results
Tutt et al. (TnT) (32)	III	Carboplatin vs Docetaxel	Metastatic	ORR	BRCAm group ORR 68% vs 33% PFS 6.4 vs 4.4 months
Byrski et al. (33)	Retrospective	Cisplatin	Neoadjuvant	pCR	pCR = 83%
Hahnen et al. (34) (GeparSixto)	II	Carboplatin vs SoC ChT	Neoadjuvant	pCR	pCR 65.4% vs 66.7%
Tung et al. (35) (INFORM)	II	Cisplatin vs Doxorubicin & Cyclophosphamide	Neoadjuvant	pCR	18% vs 26%
Robson et al. (36) (OlympiAD)	III	Olaparib vs SoC ChT	Metastatic	PFS	7 vs 4.2 months
Litton et al. (37) (EMBRACA)	III	Talazoparib	Metastatic	PFS	8.6 vs 5.6 months
Drew et al. (38)	II	Rucaparib	Metastatic	RR	15%
Dieras et al. (39) (BROCADE3)	III	Veliparib + paclitaxel carboplatin vs Placebo + paclitaxel Carboplatin	Metastatic	PFS	14.5 vs 12.6 months
Vinayak et al. (40)	II	Niraparib + pembrolizumab	Metastatic	RR	BRCAm group PR 47%

ORR, overall response rate; pCR, pathological complete response; PFS, progression free survival; RR, response rate; BRCAm, BRCA mutated; ChT, chemotherapy; SoC, standard of care.

### Platinum Salts

Since the introduction of cisplatin in the 1970s, platinum compounds have been the cornerstone in the treatment of various tumour types. Platinum agents form intra-strand adducts by binding with the purines leading to DSBs. This triggers various repair mechanisms including that of homologous recombination (HR) (41). Consequently, cells with HR deficiency can be particularly sensitive to platinum compounds (42, 43).

In a small phase II open label study, 20 BRCA1 mutation carriers with metastatic breast cancer (mBC) received cisplatin 75 mg/m2 on a 3-weekly basis with 35% achieving partial response and 45% complete response with acceptable toxicity profile (44). In the Phase II TBCR009 trial, 86 previously treated triple negative mBC patients received either cisplatin or carboplatin. Response rates in the BRCA1/2 mutation carrier patient subgroup were significantly higher compared to the total study population (54% versus 26%) (45).

The triple negative breast cancer trial (TNT) was the largest trial examining the role of platinum compounds in the treatment of triple negative and BRCA1/2 mutated mBC patients. In this Phase III study, 376 mBC patients were randomised to receive first line chemotherapy with carboplatin or docetaxel. In the BRCA1/2 mutation subgroup the overall response rates were higher for the carboplatin group (68% vs 33%). Similarly, PFS was also improved in the BRCA1/2 mutation carriers who received carboplatin (6.4 vs 4.4 months) (32).

The use of platinum compounds has also been assessed in the neoadjuvant setting. In 2010, Byrski et al. reported a pathological complete response (pCR) rate of 83% for women with BRCA1 positive BC treated with neoadjuvant cisplatin (33). This was further echoed in the findings of a single arm study including 107 BC patients carrying BRCA1 mutation who were treated with 4 cycles of neoadjuvant chemotherapy with 61% achieving pCR (46).

In GeparSixto, a phase II randomised trial, triple negative stage II-III breast cancer patients were given anthracycline and taxane based neoadjuvant chemotherapy with or without carboplatin (47). In a secondary analysis, BRCA1/2 mutation carriers did not gain any additional benefits in terms of pCR with the addition of carboplatin (65.4% vs 66.7%) with similar impact on DFS. On the contrary, carboplatin conferred significant improvement in response rates to non-carriers (34). In the phase II CALBG 40603 trial, although the addition of carboplatin to NACT achieved superior pCR rates in patients with II-III triple negative BC, an improvement in long term survival outcomes was not demonstrated (48). Results from the recent randomized Phase II INFORM trial, demonstrated that in BRCA1/2 carriers with HER2 negative stage I-III BC, neoadjuvant single agent cisplatin did not achieve better pCR compared to doxorubicin and cyclophosphamide (AC) (35). All things considered, the use of platinum compounds as part of neoadjuvant chemotherapy does not clearly improve the rates of pCR in breast cancer patients carrying BRCA1/2 mutations.

### PAPR Inhibitors

The concept that some genes can be “synthetically lethal” has been well known since early preclinical studies. In order for two genes to be synthetically lethal, both have to carry mutations leading to cell death. As a result, the targeting of one gene, combined with a known genetic mutation could be a tempting field for the development of new anticancer drugs (49).

Under this scope, the inhibition of single strand (SS) DNA repair with the use of the enzyme poly (ADP) ribose polymerase (PARP) inhibitors, in combination with known homologous recombination (HR) deficiency, can result in cell death (50).

Over the past 6 years multiple PARP inhibitors have been approved for the treatment of ovarian cancer (51). Olaparib is the PARP inhibitor which has been studied more extensively in breast cancer patients with BRCA1/2 mutations. In the early phase clinical trial olaparib showed efficacy in advanced solid tumors with 22 patients having breast cancer and 9 of them being BRCA1/2 mutant (52). In a proof of concept trial 54 pretreated metastatic breast cancer patients with BRCA1/2 mutation were treated with olaparib 400 mg twice daily (BD) or 100 mg BD. On the 400 mg BD arm, overall response rate was 41% and 22% in the cohort of 100 mg BD with acceptable toxicity profile (53). In another phase II basket trial 62 women with advanced breast cancer received olaparib. ORR was achieved in 13% of patients and stable disease for more than 8 weeks was observed in 47% (54). The ORR was lower in patients with previous exposure to platinum compounds suggesting that there is cross-resistance with PAPR inhibitors.

In the randomized open label phase III OlympiAD trial, olaparib 300 mg BD monotherapy was compared with standard chemotherapy (eribulin, capecitabine, gemcitabine) in 302 patients with metastatic, HER2 negative, BRCA1/2 related breast cancer. All patients had received anthracycline and taxane based chemotherapy. Median progression free survival was significantly improved for the olaparib arm (7 months vs 4.2 months). The response rates were 59.9% for the olaparib group and 28.8% for the chemotherapy group (36). Of note, olaparib was not compared to cisplatin or carboplatin.

Talazoparib is a potent PARP inhibitor which has been studied for the treatment of BRCA1/2 mutation related breast cancer. In the early clinical trial, talazoparib showed promising activity in BRCA1/2 mutation related solid tumors including patients with breast cancer (55). EMBRACA was a phase III open label clinical trial, which randomised 431 metastatic breast cancer patients with germline BRCA1/2 mutations to talazoparib or physician’s choice chemotherapy. Median PFS was significantly improved in the talazoparib arm (8.6 months vs. 5.6 months) (37). ABRAZO was a phase II, trial assessing the efficacy of talazoparib in germline BRCA1/2 mutant breast cancer patients with previous response to platinum-based chemotherapy or in patients with 3 or more previous lines of cytotoxic treatment and demonstrated promising anti-tumour activity (56).

Talazoparib has also been tested in the early breast cancer setting. After the promising results of a feasibility study in which 2 months of neoadjuvant treatment with talazoparib before the initiation of standard neoadjuvant chemotherapy, showed median decrease in tumor size of 88% (57), a separate pilot study was organized. Twenty patients with germline BRCA1/2 mutant HER2 negative breast cancer received 6 months of neoadjuvant treatment with talazoparib before proceeding with surgery. Pathological complete response was achieved in 53% of the patients with acceptable toxicity (58).

Another PARP inhibitor, rucaparib has been evaluated for the treatment of patients with metastatic breast cancer. In a phase II, open-label, multicentre trial of rucaparib in BRCA1/2 mutation carriers with advanced breast or ovarian cancer, the range of dosing schedules, safety and tolerability were assessed. The treatment schedule included intravenous and subsequently oral rucaparib. In the intravenous only schedule response rated was only 2%, with 15% on the continuous oral schedule. The authors concluded that in order to achieve optimal response continuous dosing schedule is required (38).

Veliparib has also been tested in germline BRCA1/2 mutation carrier breast cancer patients. In a phase II trial, veliparib was given as a monotherapy at 400 mg BD and at the time of progression carboplatin at a dose of AUC5 was added. Partial response rate was 17% for BRCA1 and 23% for BRCA2 mutation carries who had at least 4 cycles of follow-up (59).

Recently the results of phase III BROCADE3 trial were presented. In this trial 509 germline BRCA1/2 mutation carriers with metastatic breast cancer were randomised 2:1 to receive paclitaxel/carboplatin plus intermittent veliparib or paclitaxel/carboplatin plus placebo. Median PFS was improved by 1.9 months (14.5 vs 12.6 months) (39).

The results of a phase II open label trial of niraparib in combination with pembrolizumab were recently announced (40). In this study, 55 women with triple negative metastatic breast cancer were treated with niraparib at a dose of 200 mg once daily combined with pembrolizumab 200 mg every 3 weeks. Fifteen patients had somatic or germline BRCA1/2 mutation with 7 achieving partial response (47%).

There are no data to support the use of systemic treatments in patients with moderate-risk breast cancer susceptibility mutations. This is currently investigated in a Phase II clinical trial which explores the effectiveness of olaparib in mBC patients with somatic or germline mutations in DNA repair genes. Preliminary data shown efficacy in patient with somatic BRCA1/2 and germline PALB2 mutations but not in those with ATM or CHEK2 mutations (60).

## Conclusion

Treating hereditary breast cancer entails more challenges than sporadic cases. High risk patients such as BRCA1/2 germline mutation carriers, present at a young age and their optimal surgical management yet remains an individualized and debatable area. BRCA1/2 mutation carriers face more aggressive surgical interventions for therapeutic and risk reducing purposes due to their high risk of developing primary or contralateral breast cancer. Breast conserving surgery as well as skin sparing mastectomies with or without preservation of the nipple-areolar complex have been proven to be safe and achieve better cosmesis. Selecting the best surgical approach for this patient population requires taking into account several factors including patient’s genetic risk, family history, previous BC biology, as well as patient’s own preferences.

Due to defects in homologous recombination, BRCA1/2 related BC is highly susceptible to treatment with platinum compounds. Several clinical trials demonstrated higher response rates with platinum in BRCA1/2 mutation carriers with metastatic BC. However, this finding was not replicated in the neoadjuvant setting, where an additive benefit of platinum compounds in achieving pCR has not been demonstrated for BRCA1/2 mutation carriers.

The therapeutic landscape of BRCA1/2 related breast cancer has been enriched with the addition of PARP inhibitors which led to improvements in survival outcomes. Olaparib and talazoparib have already gained regulatory approval while other such as niraparib and rucaparib and veliparib are undergoing clinical trial assessment. Combinatorial strategies involving PARP inhibitors with chemotherapy or immunotherapy are also being under investigation and hold promise for the future management of BRCA1/2 related breast cancer.

## Author Contributions

All authors listed have made a substantial, direct, and intellectual contribution to the work, and approved it for publication.

## Conflict of Interest

The authors declare that the research was conducted in the absence of any commercial or financial relationships that could be construed as a potential conflict of interest.

## Abbreviations

N, number; BCT, breast conserving treatment; UBC, unilateral breast cancer; M, mastectomy; BC, breast cancer; CRRM, Contralateral risk reducing mastectomy; OS, Overall survival;CBC, contralateral breast cancer; PYO, person years of observation; LR, local recurrence; EBC, early breast cancer; BPO, bilateral salpingo-oophorectomy.
